# Robots do not judge: service robots can alleviate embarrassment in service encounters

**DOI:** 10.1007/s11747-022-00862-x

**Published:** 2022-04-20

**Authors:** Jana Holthöwer, Jenny van Doorn

**Affiliations:** Department of Marketing, University of Groningen, P.O. Box 800, 9700 AV Groningen, the Netherlands

**Keywords:** Service robots, Social judgment, Embarrassment, Automated social presence, Anthropomorphism

## Abstract

Although robots are increasingly used in service provision, research cautions that consumers are reluctant to accept service robots. Five lab, field, and online studies reveal an important boundary condition to earlier work and demonstrate that consumers perceive robots less negatively when human social presence is the source of discomfort. We show that consumers feel less judged by a robot (vs. a human) when having to engage in an embarrassing service encounter, such as when acquiring medication to treat a sexually transmitted disease or being confronted with one’s own mistakes by a frontline employee. As a consequence, consumers prefer being served by a robot instead of a human when having to acquire an embarrassing product, and a robot helps consumers to overcome their reluctance to accept the service provider’s offering when the situation becomes embarrassing. However, robot anthropomorphism moderates the effect as consumers ascribe a higher automated social presence to a highly human-like robot (vs. machine-like robot), making consumers feel more socially judged.

**Supplementary Information:**

The online version contains supplementary material available at 10.1007/s11747-022-00862-x.

That robots will be increasingly employed in frontline service settings is strongly indicated by a 61% rise in sales of robots for professional use from 2017 to 2018 (International Federation for Robotics, 2019). Challenges associated with aging populations and an ever-increasing shortage of personnel have led the healthcare sector to invest heavily in service robots (Broadbent et al., 2010), and in the hospitality sector “Pepper” the robot often greets and serves customers in restaurants, airports, and cruise ships (Blut et al., 2021; Mende et al., 2019).

Following prior research, we define robots as “information technology in a physical embodiment, providing customized services by performing physical as well as nonphysical tasks with a high degree of autonomy” (Jörling et al., 2019, p. 405). Importantly, this definition does not encompass self-service technology such as ATMs or self-checkout machines owing to their low level of autonomy.

Although reliance on service robots is rising, customers do not always readily accept robots, and their use can evoke skepticism and trigger negative feelings (Čaić et al., 2019; Mende et al., 2019). Hence, theoretical and empirical exploration is imperative to uncover how robots’ negative effects can be attenuated or perhaps avoided altogether. We investigate whether consumers are more accepting of service robots in situations where human presence causes social discomfort—particularly service settings that can create feelings of being socially judged by the human service provider. A perception of social judgment can arise owing to the product that needs to be acquired, such as condoms (Dahl et al., 2001), or through interaction with a frontline employee, as when they are confronted with their own errors or forgetfulness (Grace, 2009). In such settings, the presence of others can trigger feelings of embarrassment (Argo et al., 2005; Dahl et al., 2001) owing to a fear that negative judgment will pose a threat to one’s social identity (Edelmann, 1987; Higuchi & Fukada, 2002).

This concern leads consumers to either avoid such situations by buying products such as medication to treat a sexually transmitted disease (STD) or incontinence drugs online and to use self-service checkouts in physical stores (Krishna et al., 2019; Jackson et al., 2014) or to retreat as quickly as possible from an embarrassing situation that occurs unexpectedly (Grace, 2009; Jones et al., 2018; Krishna et al., 2015). Importantly, embarrassing service encounters can have long-term negative consequences for companies because consumers avoid the service provider in the future (Grace, 2009). In the healthcare domain, avoiding embarrassing situations can jeopardize consumers’ well-being.

We propose that being served by a robot instead of a human can mitigate the fear of being socially judged because automation removes the human social presence that triggers perception of social judgment (Argo et al., 2005; Dahl et al., 2001). The concept of automated social presence has recently been introduced and is defined as “the extent to which technology makes customers feel the presence of another social entity” (van Doorn et al., 2017, p. 43). Compared to human social presence, automated social presence should lead to lower perception of social judgment. This prediction is in line with a recent study showing that a robot reduces feelings of embarrassment because of its lower perceived agency (i.e., being able to act with intention and form opinions) (Pitardi et al., 2021). However, whether robotic service translates into a higher likelihood to acquire an embarrassing product or leads to the consumer’s greater comfort when facing an embarrassing situation is so far unclear. We expect service robots to make customers feel less judged and more at ease when acquiring an embarrassing product or being disparaged by the service employee, leading to a preference for service robots when the situation becomes embarrassing and to a higher likelihood to accept the service provider’s offering.

We also explore the extent to which automated social presence of a robot is affected by anthropomorphism in terms of having human-like features such as a face, arms, and legs. While marketing has found anthropomorphism to increase product and brand liking (Aggarwal & McGill, 2012), whether anthropomorphizing service robots enhances customer experiences is unclear (Blut et al., 2021). We hypothesize that robot anthropomorphism can trigger higher perceptions of automated social presence because the robot appears livelier and more engaging (Jung & Lee, 2004; van Doorn et al., 2017). While this effect may be desirable in many situations, robot anthropomorphism and the related heightened automated social presence may backfire when consumers fear social judgment. In these situations, consumers may perceive a highly anthropomorphic robot as more judgmental than a machine-like robot and therefore may be more reluctant to acquire an embarrassing product and more likely to avoid a service provider after an embarrassing encounter. Previous literature has indeed found patients to be less embarrassed during a medical examination when instructed by a technical box than by a robot with human-like features (Bartneck et al., 2010). Further, more human-like conversational agents elicit more socially desirable responses, and less honest answers, to sensitive questions (Schuetzler et al., 2018). We propose that these effects may generalize to embarrassing situations in domains beyond healthcare, such as hospitality services, and to interactions with robots with different levels of human likeness.

Our investigation makes three contributions to the literature. First, we show a key boundary condition to earlier work cautioning that consumers are reluctant to accept service robots (Čaić et al., 2019; Mende et al., 2019). Importantly, we find that consumers are more accepting of service robots in situations where they fear human social judgment. We investigate an array of managerially relevant attitudinal and behavioral outcomes, such as the propensity to acquire an embarrassing product and likelihood to accept an alternative offering from the same provider. Second, we show the underlying process driving this effect. Consistent with social identity theory (Edelmann, 1987; Miller & Leary, 1992), consumers feel less socially judged by a robot than by a human, resulting in a higher likelihood they will accept the service provider’s offerings. We link the literature on embarrassment (Dahl et al., 2001; Grace, 2009; Jones et al., 2018) and social identity theory (Edelmann, 1987; Miller & Leary, 1992) to the increasingly important research field of service robots and technology (Huang & Rust, 2017, 2018; Kumar et al., 2016; van Doorn et al., 2017; Wirtz et al., 2018). Third, we demonstrate the critical role of a service robot’s design in shaping perceptions of automated social presence, in particular the robot’s level of anthropomorphism. We show that a more anthropomorphic robot is associated with greater automated social presence, making people feel more judged and hence increasing their tendency to avoid dealing with a company when served by a highly human-like service robot.

## Theoretical framework

### Service robots

The increasing use of service robots to automate frontline interactions by executing simple, standardized tasks and delivering services in a reliable, fast, and efficient way is reflected in the emerging literature stream on service robots (Huang & Rust, 2018, 2020; Wirtz et al., 2018). While current research on service robots is largely conceptual, we review empirical studies on the role of service robots across a number of service settings. Table 1 gives a non-exhaustive overview of the research into the acceptance and evaluation of service robots and related relevant issues, such as robot anthropomorphism.

**Table 1:** Research on the acceptance of service robots

Paper	Method / Setting	Robot type	Compared to human	Dependent variable	Effect of robot (+ or -)	Findings
Acceptance of robots						
Čaić et al. (2018)	Interviews	Humanoid	No		+ / -	Robots can have three support roles in elder care (i.e., physical, psychosocial, and cognitive health), which may contribute more to the individual (extended self), the in/formal network of caretakers (replacement), or the individual and network (enabler, intruder).
Čaić et al. (2019)	Field experiments	Humanoid	Yes	Intentions to use	–	Elderly patients perceived robotic (vs. human) coaches as less warm and less competent, lowering behavioral intentions to participate in exercise games.
Huang and Rust (2018)	Conceptual paper		Yes			Development of a theory of AI job replacement which specifies four intelligences required for service tasks—mechanical, analytical, intuitive, and empathetic. Lays out how firms should decide between humans and AI for accomplishing those tasks.
Jörling et al. (2019)	Interviews, online experiments	Non-humanoid	No	Outcome responsibility	+ / -	A service robot’s autonomy decreases perceived behavioral control over it, which decreases perceived responsibility for positive service outcomes but not for negative service outcomes. Customers feel responsible for negative service outcomes if they perceive ownership of the robot. Customer’s potential to interrupt the service robot’s autonomy increases perceived control and perceived responsibility for positive outcomes.
Merkle (2019)	Lab experiment	Humanoid	Yes	Customer satisfaction	+	After a service failure, customer satisfaction is greater with a service robot compared to a human service provider.
Pitardi et al. (2021)	Interviews, online experiments	Humanoid	Yes	Anticipated embarrassment		Embarrassment during a potentially embarrassing encounter is lower when interacting with a service robot (vs. human employees) due to lower perceived agency.
Smarr et al. (2012)	Interviews	Humanoid	Yes	Acceptance of robots, assistance preference	+ / -	Older adults preferred robot assistance for performing instrumental (e.g., housekeeping) and enhanced activities (e.g., hobbies) but preferred human assistance in performing activities of daily living (e.g., prepare meals).
Van Doorn et al. (2017)	Conceptual paper		Yes	Customer and service outcomes	+	The concept of automated social presence (ASP) is defined and related to several key service and customer outcomes, mediated by social cognition and perceptions of psychological ownership as well as three customer-related factors that moderate the relationship between ASP and social cognition and psychological ownership (i.e., tendency to anthropomorphize, a customer’s relationship orientation, and technology readiness).
Wirtz et al. (2018)	Conceptual paper		No			The authors define service robots and outline for which types of service tasks robots versus humans will dominate. Consumer perceptions, beliefs and behaviors as related to service robots, and advances the service robot acceptance model are examined. An overview of the ethical questions surrounding robot-delivered services at the individual, market and societal level is given.
Robot anthropomorphism						
Bartneck et al. (2010)	Lab experiment	Various	No	Posture shift, head away, gaze shift, nervous smile, gaze down, face touch, hand movement, situation embarrassment	+	Participants were less embarrassed when interacting with a technical box (vs. a technical robot and a lifelike robot) in a medical examination.
Kim et al. (2019)	Online experiments	Various	Yes	Consumer attitudes	–	Anthropomorphism of a consumer robot increases psychological warmth but did not significantly affect competence judgments. Uncanniness mediated the relationship between warmth and liking of the robot.
Mende et al. (2019)	Online and lab experiments	Various	Yes	Compensatory behaviors	–	Humanoid service robots increase compensatory behavior owing to increasing discomfort. Social belongingness and healthiness of food moderate this effect. A less anthropomorphic robot (i.e., machine-like) buffers the increase in food consumption.
Van Pinxteren et al. (2019)	Field experiment	Humanoid	No	Intentions to use	+	Gaze cues increase anthropomorphism when comfort is low and decrease it when comfort is high. Anthropomorphism of a service robot has a positive effect on consumers’ perceived trust. Mediation occurs such that consumers’ perceived trust positively influences perceived enjoyment, which in turn has a positive effect on consumers’ intentions to use humanoid service robots.
Current research						
	Online, lab and field experiments	Various	Yes	Choice robot vs. human, accept (vs. not accept) a service provider’s offering	+	A service robot (vs. human service provider) makes consumers feel less judged when having to face an embarrassing service situation. Consequently, service robots help overcome consumer reluctance to acquire embarrassing products. Robot anthropomorphism moderates the effect such that a highly human-like robot (vs. a machine-like robot) triggers perceptions of automated social presence and higher social judgment.

**Fig. 1:**
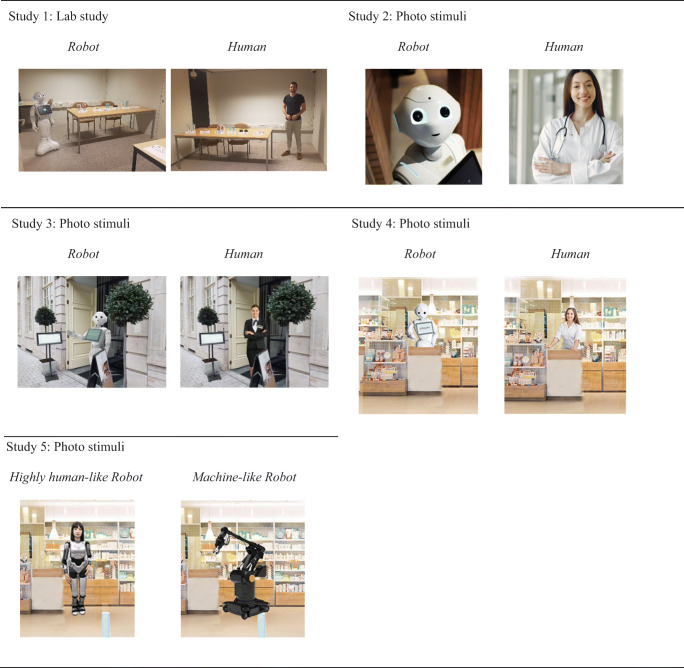
Illustrative photos of the service providers used across the studies

Empirical evidence so far is mixed regarding whether consumers accept or resist service by robots. For example, replacing human employees with service robots can result in negative service outcomes. In hospitality services, interacting with a service robot rather than a human employee can trigger feelings of eeriness and human identity threats, leading to consumers’ compensatory behaviors such as status consumption and increased food intake (Mende et al., 2019). The elderly rated a robotic coach lower than a human coach on warmth and competence and as a consequence were less likely to participate in exercise games (Čaić et al., 2019). However, after a service failure, consumers may evaluate a service robot more favorably than a frontline human service employee (Merkle, 2019), and robots have been found to reduce embarrassment (Pitardi et al., 2021).

Other research has highlighted that preference for a robot over a human can be task-specific. For instance, older people preferred robotic to human assistance for some instrumental activities such as housekeeping or setting medication reminders but not for other common daily living tasks, such as shaving, walking, or getting dressed (Smarr et al., 2012). Older people also perceived socially assistive robots as having a positive impact on co-creating service value (e.g., as an extended self, an enabler, and an ally) but also as having co-destruction potential (e.g., as a deactivator, a replacement, and an intruder) (Čaić et al., 2018).

Studies have also investigated the impact of robot design, which often varies to match the service context and the characteristics of the consumer (Lu et al., 2020). One important element affecting robot acceptance is the degree of anthropomorphism, which is inherently required for a robot’s ability to engage in meaningful interactions (Duffy, 2003). Anthropomorphizing objects means imbuing the objects with “the real or imagined behavior of nonhuman agents with human-like characteristics, motivations, intentions, or emotions” (Epley et al., 2007, p. 864). One perception is that consumers respond positively to robots with human-like behavioral characteristics because they can relate easily to them and bond with them (Broadbent et al., 2013), and research results have highlighted the favorable impact of anthropomorphizing service robots on customers’ trust, intention to use, and enjoyment (van Pinxteren et al., 2019).

However, another school of thought cautions that robot anthropomorphism can backfire, often building on the uncanny valley theory (Mende et al., 2019; Mori et al., 2012). This theory proposes that amplifying human-likeness increases affinity for the robot until the robot closely resembles a human. Then, a strong feeling of uncanniness occurs, resulting in a radical shift from positive to negative responses because a robot that looks human often cannot enact the expected human behavior (Mori et al., 2012). Empirical research shows that, as predicted by the theory, anthropomorphizing a robot increases psychological warmth up to a certain point, where it decreases liking owing to a feeling of uncanniness (Kim et al., 2019). In some cases, making a robot less anthropomorphic decreases negative consumer responses (Mende et al., 2019). For example, a technical box that gave instructions during a medical examination elicited less embarrassment than an anthropomorphic robot (Bartneck et al., 2010). Research into anthropomorphism in human–robot interaction has also examined other robot features, such as autonomy (Kahn et al., 2006). In one study, perceived autonomy of a service robot led to customers feeling lower levels of control and responsibility for positive outcomes (Jörling et al., 2019).

Overall, previous research has yielded mixed findings regarding the acceptance of service robots and the role of robot anthropomorphism. Therefore, exploration of potential boundary conditions affecting the extent to which consumers accept robots serving them is important. Furthermore, the literature has so far largely linked anthropomorphism to feelings of uncanniness and has not empirically examined whether robot anthropomorphism also affects the automated social presence ascribed to a robot.

### Embarrassment and social judgment

Previous work has investigated different types of embarrassing situations, like being the center of attention or committing a faux pas (Sabini et al., 2001). Embarrassment can occur in various service settings, such as during purchase of products like body care or sex-related products (Jones et al., 2018; Krishna et al., 2015; Lau-Gesk & Drolet, 2008) or in some use situations, such as when the credit card is denied while paying the bill.

Ample evidence shows that human social presence plays a central role in determining consumer behaviors and attitudes (Argo et al., 2005; Dahl et al., 2001). While social presence may enhance service satisfaction when the service experience is positive (He et al., 2012), scholars have also emphasized potential downsides of social presence (Esmark et al., 2017; Grewal et al., 2020). One drawback is that the real or imagined social presence of others can result in consumers feeling socially judged in embarrassing situations. This perception leads consumers to avoid purchasing embarrassing products, withdraw as quickly as possible from the embarrassing situation, or avoid the service provider for future transactions (Argo et al., 2005; Dahl et al., 2001; Grace, 2009).

The social evaluation model posits that embarrassment is caused by the feeling of being negatively or undesirably evaluated by others (Manstead & Semin, 1981; Miller, 1996; Semin & Manstead, 1981). As people have a concern for how they are appraised by others, negative social judgments can evoke a threat to their social identity (Edelmann, 1987; Miller & Leary, 1992) and influence patronage decisions (Grace, 2009). This social identity threat corresponds with the premise of social identity theory that an individual strives to achieve a satisfactory image of the self.

Previous work has shown that specific frontline employee behaviors, such as criticizing a customer, can pose a threat to a customer’s social identity (Habel et al., 2017). Consumers go to great lengths to avoid social judgment by others and protect their social identity, endangering their health, engaging in risky behavior, and avoiding medical care even when major health problems are present (Helweg-Larsen & Collins, 1994; Kiefe et al., 1998). Research findings suggest that having to shop for a sensitive product that others may not approve of can be perceived as a threat to social identity, provoking feelings of social judgment when others are present. When making an embarrassing purchase, consumers visit less-crowded stores, avoid asking employees for help, and wait for other customers to leave the aisle (Blair & Roese, 2013). More recently, consumers have started to shop online for items such as dieting products or plus-size clothing and to opt for using self-service checkout systems when, for example, purchasing condoms (Krishna et al., 2019; Jackson et al., 2014).

However, online purchases and self-service checkouts can pose difficulties through technological complexity or temporal issues (i.e., the product not being immediately accessible via the internet) (Miyazaki & Fernandez, 2001; Young et al., 2017). In addition, use of self-serving technologies and online retailers may raise legal, ethical, and safety issues, as when buying medications in pharmacies (Fung et al., 2004). Importantly, these strategies do not work when the source of embarrassment lies in social interaction between a frontline employee and a customer (Miller & Leary, 1992). While consumers can at least mentally prepare themselves when having to acquire an embarrassing product, interactional situations often occur unexpectedly, such as when a service employee criticizes customers or confronts them with their own errors (Grace, 2007, 2009).

Building on literature showing that human social presence in service interactions can elicit negative feelings (Dahl et al., 2001; He et al., 2012), we expect these discomforting feelings to decrease when social presence is automated through use of a service robot in place of a human service provider. In these situations, the automated service interaction may reduce a consumer’s apprehension of social judgment (Higuchi & Fukada, 2002), a prediction in line with a study showing that a robot reduces feelings of embarrassment (Pitardi et al., 2021). This reduction should make it easier to face a situation such as acquiring embarrassing products or facing criticism by a frontline employee. As a consequence, consumers prefer robot service providers over human ones in embarrassing situations, and are more likely to accept the service provider’s offering instead of retreating as quickly as possible. We propose:

- **H1** In a more (vs. less) embarrassing service encounter, consumers are more likely (a) to prefer a robot over a human service provider, and (b) to accept a service provider’s offering when served by a robot (vs. a human service provider).
- **H2** The effect of the robot (vs. human) service provider on consumers’ likelihood to accept a service provider’s offering is mediated by social judgment such that consumers feel less socially judged by a service robot (vs. a human service provider) in an embarrassing situation.

## Empirical overview

We test our hypotheses in a series of five experiments (Table 2). Study 1, a laboratory study, shows that when having to acquire an embarrassing product through an in-person encounter with a human or an encounter with a robot service provider, consumers prefer the robot over the human service provider.^1^ Study 2, a field study, comprises a real advertising campaign on Facebook which shows that users are more likely to click on an ad promoting an embarrassing service when the ad features a robot rather than a human service provider.^2^ Studies 3–5 were conducted online through the platform Prolific.^3^ Study 3 shows that consumers are more likely to accept an alternative offering from a robot (vs. human) when confronted with their own error. Study 4 sheds light on the underlying process and establishes that consumers feel less socially judged by a robot and therefore are more likely to acquire an embarrassing product in a pharmacy. Study 5 empirically tests a third hypothesis on the role of robot anthropomorphism. We show that consumers feel less judged by a machine-like robot than by a highly human-like robot, owing to differences in perceived automated social presence, affecting the likelihood the consumer will acquire an embarrassing product.

**Table 2:** Overview of studies

Study	Design and stimuli	Response type	Dependent variable	Findings	Hypotheses
S1	2 (embarrassment: low vs. high) between-subjects, lab experiment	Actual behavior	Choice of service provider	Participants were more likely to choose the service robot when having to acquire the highly embarrassing product (Choice_robot_ = 75%, Choice_human_ = 25%), while they were more likely to choose the human service provider when having to acquire the less embarrassing product (Choice_robot_ = 32%, Choice_human_ = 68%; Wald χ2 = 15.82, *p* < 0.001, η2 = 0.43).	H1a supported
S2	2 (robot, human) × 2 (embarrassment: low vs. high) between-subjects, field study	Actual behavior	Click-through rates (CTR)	CTR for the more embarrassing ad higher for robot (1.26%) than human counselor (0.74%; Wald χ2 = 4.37, *p* = 0.037, η2 = 0.03). No significant difference for the less embarrassing ad (robot 0.93% and human 0.73%; Wald χ2 = 0.81, *p* = 0.37).	H1b supported
S3	2 (robot, human) × 2(embarrassment: low vs. high) between-subjects, photo stimuli	Intentions	Likelihood of accepting an alternative offering	Engaging with a service robot during an embarrassing service encounter makes consumers more likely to accept an alternative offering from the same service provider (vs. human) (robot = 3.72, human = 3.48; *F*(1,376) = 4.96, *p* = 0.027).	H1b supported
S4	2 (robot, human) × 2 (embarrassment: low vs. high) between-subjects, photo stimuli	Intentions	Intentions to acquire the product	The intention to acquire an embarrassing product is higher with a service robot (vs. human service provider) (robot = 5.48, human = 5.15; *F*(1,401) = 5.38, *p* = 0.021). Mediation via social judgment.	H1b supported H2 supported
S5	2 (highly human-like robot vs. machine-like robot) × 2 (embarrassment: low vs. high) between-subjects, photo stimuli	Intentions	Intentions to acquire the product	Perceived ASP is higher for a highly human-like service robot (vs. a machine-like), which in turn led to higher social judgment and a lower the intention to acquire the embarrassing product (highly human-like = 5.15, machine-like = 5.56; index of moderated mediation = −0.05, SE_*b*_ = 0.02, 95% CI [−0.10, −0.001]).	H3 supported
NR1	2 (robot, human) × 2 (embarrassment: low vs. high) between-subjects, video stimuli	Actual behavior	Choice of taking (vs. not taking) a product	Participants were more likely to choose the embarrassing (vs. less embarrassing) product with a robot (vs. a human) (robot = 61%, human = 38%, Wald χ^2^ = 9.30, *p* = 0.002).	H1b supported
NR2	2 (robot, human) × 2 (embarrassment: low vs. high) between-subjects, photo stimuli	Intentions	Loyalty intentions	Encountering a robot (vs. a human service employee) makes customers more loyal to a service provider after an embarrassing service episode (robot = 4.12, human = 3.80; *F*(1,381) = 5.34, *p* = 0.021). Mediation via social judgment.	H1b supported H2 supported
NR3	3 (highly human-like robot vs. machine-like robot vs. human) × 2 (embarrassment: low vs. high) between-subjects, photo stimuli	Intentions	Intentions to acquire the product	The intention to acquire an embarrassing product is higher with a machine-like robot (vs. human) and equally high for the highly anthropomorphic robot and human (highly human-like = 5.58, machine-like = 6.03, human = 5.75; *F*(2,1065) = 27.85, *p* < 0.001).	H3 supported

NR = not reported; ASP = automated social presence

Illustrative photos of the service providers used across the studies appear in Fig. 1. Details of all instructions, manipulations, and measures used in our studies, as well as further analyses of our data including robustness checks, appear in the Web Appendix.

## Study 1

Participants were 104 students who took part in a laboratory study for money or course credit. Six participants were excluded from the analysis because they did not read the instructions properly, leaving a final sample of 98 (51 males, M_age_ = 20.94, SD_age_ = 3.64). The study employed a two-cell (embarrassment: high vs. low) between-subjects design.

### Pretest of the stimuli

We pretested our manipulation with a sample of 200 Dutch students recruited from Prolific (76 males, M_age_ = 21.77, SD_age_ = 2.92) in a scenario-based study with a 2 cell (embarrassment: high vs. low) between-subjects design. We asked respondents to indicate how embarrassed, uncomfortable, ashamed, and awkward they would feel to acquire each of either three more embarrassing products (a chlamydia self-test, a sex toy, and hemorrhoid cream) or less embarrassing products (a COVID-19 self-test, a fitness massage ball, and hand cream) (Cronbach’s α = 0.92–0.97; 1 = “strongly disagree” and 7 = “strongly agree”; Dahl et al., 2001). Afterwards, respondents ranked which product would be most embarrassing to buy (high embarrassment condition) or least embarrassing to buy (low embarrassment condition).

An analysis of variance (ANOVA) revealed that the highly embarrassing products were indeed perceived as more embarrassing (M_STD self-test_ = 4.25, M_hemorrhoid cream_ = 4.26, M_sex toy_ = 4.90) than the less embarrassing products (M_COVID-19 self-test_ = 2.53, M_hand cream_ = 1.91, M_fitness massage ball_ = 2.27; *F*(1,198) = 170.38, *p* < 0.001). Furthermore, participants felt significantly more embarrassed about the product they ranked as most embarrassing (M = 5.42) than about the product ranked as least embarrassing (M = 1.23; *F*(1,198) = 876.30, *p* < 0.001).

### Procedures

In our main study, we showed participants either the three highly or the three less embarrassing products and asked them to indicate their level of embarrassment employing the same scale as in the pretest (Cronbach’s α = 0.89–0.95). Afterwards, respondents were asked to rank the products according to how embarrassed they would be to buy those. They were then instructed to acquire the product they ranked as most embarrassing (high embarrassment condition) or least embarrassing (low embarrassment condition).

Respondents were told that they could get a free sample of the chosen product at two locations, with either a human service provider or a service robot present. As locations, we used two rooms with a table displaying the products, together with paper bags to wrap the product in. Participants were told that neither service provider would know which product had to be acquired and therefore the participant would need to state it. Once respondents entered the location, the service provider said the following:

Hello, which is the product you have to acquire? Please take it now from the table. Next to the door you will find paper bags. Take one to carry the product back to your cubicle and continue with the study. Thank you.

We carefully instructed our student assistant who served as the human condition to use identical wording, speak in a monotone, and maintain a neutral facial expression to ensure comparability with the robot. Control variables (age, gender, education, and COVID-19 concerns) were measured at the end of the survey.

### Results

#### Manipulation check

An ANOVA revealed that participants felt significantly more embarrassed about the product ranked as most embarrassing (M = 5.21) than about the product ranked as least embarrassing (M = 1.32; *F*(1,96) = 604.55, *p* < 0.001). In the high embarrassment condition, 37.5% of the participants ranked the hemorrhoid cream as most embarrassing (M = 4.75), 33.3% the sex toy (M = 4.22), and 29.2% the STD self-test (M = 4.53). In the low embarrassment condition, 60% of the participants ranked the hand cream as least embarrassing (M = 1.56), 22% the COVID-19 self-test (M = 2.46), and 18% the fitness massage ball (M = 1.98).

#### Choice of service provider

We performed a binary logistic regression with type of product as independent variable and choice of service provider as dependent variable, controlling for age, gender, and COVID-19 concerns. Results show a significant effect of type of product: participants were more likely to choose the service robot when having to acquire the highly embarrassing product (Choice_robot_ = 75%, Choice_human_ = 25%), whereas they were more likely to choose the human service provider when having to acquire the less embarrassing product (Choice_robot_ = 32%, Choice_human_ = 68%; Wald χ^2^ = 15.82, *p* < 0.001, η^2^ = 0.43).

### Discussion

Study 1 involved actual human–robot interaction and shows that respondents were more likely to choose the service robot when acquiring a highly (vs. less) embarrassing product: a higher proportion of respondents chose to acquire the embarrassing product from the service robot rather than the human service provider, supporting H1a. In contrast, in line with previous findings that people respond more positively to human than to robot service providers (e.g., Mende et al., 2019), participants were more likely to choose the human service provider when having to acquire a less embarrassing product.

## Study 2

We randomly exposed 11,815 Facebook users (5220 males, mean age not disclosed) to one of four advertisements (a 2 (service provider: robot vs. human) × 2 (embarrassment: high vs. low) between-subjects design). The ads were displayed on the Facebook news feed as sponsored posts saying “Check your fat percentage! Consult our robot advisor to prevent overweight and obesity (high embarrassment condition) / to assess your fitness and nutritional diet (low embarrassment condition). Have a chat online about your health and body composition.”

### Pretest of the stimuli

We pretested our advertisement with a sample of 101 U.K.-based respondents recruited from Prolific (27 males, M_age_ = 40.15, SD_age_ = 12.88) in a scenario-based study with a 2 cell (embarrassment: high vs. low) between-subjects design. In the high embarrassment condition, participants were told to imagine that they are struggling with overweight and have recently read about the potential negative consequences of obesity. In the low embarrassment condition, participants were told to imagine that they are trying to live a healthy lifestyle and have recently read about the positive effects of a nutritional diet and regular exercise. Respondents reported their embarrassment on a four-item scale (Cronbach’s α = 0.96; 1 = “strongly disagree” and 7 = “strongly agree”; Dahl et al., 2001). Participants then saw advertisements about checking the fat percentage to prevent overweight and obesity (high embarrassment condition) or to assess fitness and nutritional diet (low embarrassment condition), where they could indicate their preference for a robot versus human counselor. An ANOVA revealed that participants felt significantly more embarrassed to talk with someone about their overweight and obesity (M = 4.54) than about fitness performance issues (M = 3.47; *F*(1,99) = 9.07, *p* = 0.003).^4^

### Field study

We targeted Facebook users located in the U.K. but did not restrict participants on the basis of any other demographic factors. The ads were shown for four days in total (all ad specifications can be found in the Web Appendix). If users clicked on the ad, they were redirected to a page explaining that the ad was part of an academic research project. We used the click-through rate (CTR) as the dependent variable. CTR is a commonly used digital marketing metric that shows the number of clicks relative to the number of times the ad was displayed.

### Results

We computed the CTR for each condition and analyzed the differences in CTR across conditions. The CTR for the embarrassing ad featuring the robot (1.26%) is significantly higher than CTR for the embarrassing ad featuring the human (0.74%; Wald χ2 = 4.37, *p* = 0.037, η2 = 0.03). This range of CTRs is comparable to the rates in other recent research using Facebook advertising field studies (Kupor & Laurin, 2020). The difference in CTR between robot (0.93%) and human (0.73%) for the less embarrassing ad is not significant (Wald χ2 = 0.81, *p* = 0.37).

### Discussion

Study 2 corroborates the results of Study 1: in a field study setting on social media, consumers may prefer a robot over a human when the topic is embarrassing. Results showed that a robot (vs. human service provider) was more effective at generating clicks with social media advertising for an embarrassing health-related service, supporting H1b. This result has implications for real-world behavior because consumers tend to endanger their health and avoid medical care even when health issues are present (Helweg-Larsen & Collins, 1994; Kiefe et al., 1998). Robotic advisors may be a viable option for consumers who tend to avoid consulting health practitioners out of embarrassment.

## Study 3

Study 3, which was set in a hospitality context, examined a situation where embarrassment arises from an error on the side of the consumer (Grace, 2009). We recruited 405 participants on Prolific and excluded 25 owing to a failed attention check, leaving a final sample of 380 participants (221 males, M_age_ = 25.54, SD_age_ = 8.22) for a study with a 2 (service provider: robot vs. human) × 2 (embarrassment: high vs. low) between-subjects design. Respondents were asked to imagine going out for dinner and encountering a host at the restaurant’s entrance who welcomes guests. They are told that they cannot enter, either because they forgot to make a reservation (a highly embarrassing service situation resulting from an error on the side of the consumer) or because the restaurant is full (a less embarrassing service situation). They subsequently reported their embarrassment on a four-item scale (the same as that used in Studies 1 + 2) (Cronbach’s α = 0.89). Respondents then imagined standing in front of the restaurant, which was shown in the photo. We manipulated service provider type by showing the host as either a human or a humanoid robot (“Pepper”) (see Fig. 1).

As the dependent variable, we assessed participants’ likelihood to accept an alternative offer from the service provider and eat at another restaurant owned by the same owners, measured on a four-item scale (e.g., “Eating at the other restaurant seems to be a good alternative”; Cronbach’s α = 0.83; 1 = “strongly disagree” and 7 = “strongly agree”).

### Results

#### Manipulation check

An ANOVA revealed that participants felt significantly more embarrassed when they were not allowed to enter the restaurant because they did not have a reservation (M_high_ = 4.55) than when the restaurant was full (M_low_ = 3.70; *F*(1,379) = 26.919, *p* < 0.001).

#### Likelihood to accept alternative offering

An ANOVA revealed that participants would rather eat at the other restaurant of the same owners after they had faced a less (vs. more) embarrassing service encounter (M_high_ = 3.64, M_low_ = 3.82; *F*(1,376) = 5.38, *p* = 0.021). The main effect of service provider (robot vs. human) on the likelihood to accept the alternative offering was not significant (*p* = 0.426). The critical two-way interaction between service provider and type of situation (*F*(1,379) = 7.14, *p* = 0.008, η_p_^2^ = 0.02) indicated that participants were more likely to accept the alternative offering recommended by the robot (M_robot_ = 3.77) than that of the human when denied entrance because no reservation had been made (M_human_ = 3.50; *F*(1,376) = 6.24, *p* = 0.013, η_p_^2^ = 0.02). However, results showed no difference between the hosts when participants were not allowed to enter because the restaurant was full (M_robot_ = 3.75, M_human_ = 3.89; *p* = 0.194). When denied entrance by the human host, participants were less likely to dine at the other restaurant when facing a more (vs. less) embarrassing service encounter (M_high_ = 3.50, M_low_ = 3.89; *F*(1,376) = 12.12, *p* = 0.001, η_p_^2^ = 0.03). This choice was not the case for the service robot (M_high_ = 3.77, M_low_ = 3.75; *p* = 0.800).

### Discussion

Study 3 adds to the previous studies by examining another managerially relevant dependent variable, namely the likelihood to accept an alternative offering from the same service provider. We find that if a service robot (vs. a human) denies entrance and reminds consumers of their own forgetfulness, consumers are less likely to show the typical avoidance reaction documented in previous literature (Grace, 2009) and are instead more open to accept alternatives from the same service provider. This finding supports H1b.

## Study 4

Study 4 is set in a pharmacy context and examined whether the intention to acquire an embarrassing product differs when consumers encounter a robot (vs. human service provider). It also sheds light on the underlying process and examines the mediating role of social judgment. Using Prolific, we recruited 410 participants and excluded three owing to a failed attention check. The final sample comprised 407 participants (226 males, M_age_ = 27.48, SD_age_ = 9.32) for a study with a 2 (service provider: robot vs. human) × 2 (product embarrassment: high vs. low) between-subjects design. Respondents were asked to imagine getting a prescription from a doctor to buy antibiotics for either a sexually transmitted disease (highly embarrassing product) or an ear infection (less embarrassing product) and to subsequently report their embarrassment (see Study 1; Cronbach’s α = 0.95). Respondents then imagined standing in front of the pharmacy, which was shown in the photo. We manipulated service provider type by showing either a humanoid robot (“Pepper”) or a human in the pharmacy (see Fig. 1).

As the dependent variable, we assessed participants’ intention to acquire the medicine, measured on a four-item scale (e.g., “How likely are you to get your antibiotics at this pharmacy instead of going to another pharmacy?”; Cronbach’s α = 0.83; 1 = “strongly disagree” and 7 = “strongly agree”). Our measure of social judgment (e.g., “I was concerned that I was being evaluated in an undesirable way by others”; Cronbach’s α = 0.95; 1 = “strongly disagree” and 7 = “strongly agree”) was adapted from previous research using a 6-item Likert scale (Manstead & Semin, 1981; Miller, 1996; Semin & Manstead, 1981).

### Results

#### Manipulation check

An ANOVA revealed that participants felt significantly more embarrassed when acquiring antibiotics against an STD (M_high_ = 4.73) than antibiotics against an ear infection (M_low_ = 1.90; *F*(1,406) = 460.59, *p* < 0.001).

#### Intention to acquire the medicine

An ANOVA revealed a significant main effect: participants’ intention to acquire the medicine was higher when they sought to acquire the less embarrassing product (M_high_ = 5.31, M_low_ = 5.59; *F*(1,401) = 7.93, *p* = 0.005). The main effect of service provider was marginally significant, *p* = 0.062). The critical two-way interaction between service provider and product type was also significant (*F*(1,405) = 26.89, *p* < 0.001, η_p_^2^ = 0.06).

Simple effects show that participants had stronger intentions to acquire a less embarrassing product at the pharmacy when a human service provider was present rather than a robot (M_robot_ = 5.24, M_human_ = 5.94; *F*(1,401) = 25.46, *p* < 0.001, η_p_^2^ = 0.06). However, the effect flipped for acquisition of a highly embarrassing product: participants encountering a robot in the pharmacy had greater intentions to acquire the embarrassing product than participants encountering a human (M_robot_ = 5.48, M_human_ = 5.15; *F*(1,401) = 5.38, *p* = 0.021, η_p_^2^ = 0.01). When confronted with a human service provider in a pharmacy, participants were less likely to buy the embarrassing product (M_high_ = 5.15) than the less embarrassing product (M_low_ = 5.94; *F*(1,401) = 32.26, *p* < 0.001, η_p_^2^ = 0.07). This was not the case for the service robot (M_high_ = 5.48, M_low_ = 5.24; *F*(1,401) = 2.79, *p* = 0.096).

#### Mediation by social judgment

An ANOVA revealed that participants felt more judged by the human than by the robot (M_robot_ = 2.49, M_human_ = 3.10; *F*(1,403) = 21.56, *p* < 0.001) and when acquiring the embarrassing product (M_high_ = 3.44, M_low_ = 2.16; *F*(1,403) = 93.64, *p* < 0.001). We also find the critical two-way interaction between service provider and product type to be significant (*F*(1,406) = 29.37, *p* < 0.001, η_p_^2^ = 0.07). Contrasts showed that those acquiring the embarrassing product felt significantly less socially judged when they encountered a robot (M_robot_ = 2.77) instead of a human service provider (M_human_ = 4.10; *F*(1,403) = 49.52, *p* < 0.001, η_p_^2^ = 0.11), whereas no difference occurred for the less embarrassing product (M_robot_ = 2.21, M_human_ = 2.10; *F*(1,403) = 0.31, *p* = 0.579).

The moderated mediation analysis (Model 7; Hayes, 2015) revealed a conditional indirect effect of service provider on intentions to buy the medicine through social judgment when acquiring the embarrassing product (95% CI [0.21, 0.49]), but not when acquiring the less embarrassing product (95% CI [−0.13, 0.05]). Thus, as expected, consumers felt less socially judged by a robot than a human, which drove their intentions to buy the embarrassing product.

### Discussion

Study 4 not only corroborates our finding that consumers are more likely to acquire an embarrassing product from a robot than from a human service provider (supporting H1b), but also sheds light on the underlying process and reveals that consumers feel less judged by a robot than by a human when having to acquire an embarrassing product, supporting H2. In line with previous literature, we find that consumers prefer human service providers to robotic providers in a less embarrassing situation (Čaić et al., 2019; Mende et al., 2019).

## The role of robot anthropomorphism

Previous literature has shown that robot anthropomorphism—designing a robot to look like a human—can be a double-edged sword. While anthropomorphic robots are more trusted and are more enjoyable to interact with than their less anthropomorphic counterparts, they can elicit feelings of threat and uncanniness (Blut et al., 2021; Kim et al., 2019; Mende et al., 2019; van Pinxteren et al., 2019). Unclear is whether anthropomorphism also alters the automated social presence that is ascribed to a robot. Since a robot’s automated social presence reflects the extent to which it is perceived as a social entity (van Doorn et al., 2017), automated social presence is also likely to affect consumers’ perceptions of a robot’s capacity to socially judge. However, to the best of our knowledge, studies so far have not connected a robot’s anthropomorphic appearance to its social presence.

We expect that consumers ascribe greater automated social presence to more anthropomorphic robots. First, research has shown that a robot with more human-like social abilities is perceived as having greater social presence than a robot that behaves less like a human (Heerink et al., 2008). Second, related research on robot (dis)embodiment has shown that people react differently to embodied robots than to their non-embodied counterparts. In particular, physical embodiment (interacting with the actual social robot vs. the virtual robot shown in a video) yields higher social presence and a more positive evaluation of the robot (Jung & Lee, 2004). Similarly, participants put more effort into a negotiation game when they interacted with a physically embodied robotic character than with a disembodied screen character (Bartneck, 2003). Other research found that participants felt a greater physical presence with the robot present in the experiment room rather than being projected onto a screen (Kiesler et al., 2008). Taken together, these studies show that a robot’s appearance likely shapes perceptions of automated social presence.

While increased perceived automated social presence of the robot may be desirable in many situations, such as for encouraging customers to develop trust in the service employee (Wirtz et al., 2018), we argue that an automated presence may backfire in embarrassing service situations, particularly when consumers fear social judgment (Dahl et al., 2001; Miller & Leary, 1992). While perception of social judgment is lower with robots, we expect the extent to which consumers may feel socially judged by a robot to be malleable and subject to robot anthropomorphism. In particular, we expect service robots to be perceived as more judgmental when their automated social presence takes a more anthropomorphic form.

- **H3** In a more (vs. less) embarrassing service encounter, consumers are more likely to accept an offering from a machine-like service robot than from a highly human-like service robot owing to perceived lower automated social presence and lower social judgment.

## Study 5

Study 5 aims to establish the moderating role of robot anthropomorphism through automated social presence. Through Prolific, we recruited 709 participants and subsequently excluded six participants who failed the attention check (398 males, M_age_ = 27.05, SD_age_ = 8.99). The study employed a 2 (service robot: highly human-like robot vs. machine-like robot) × 2 (product embarrassment: high vs. low) between-subjects design. We used the lifelike robot “*Jia Jia*” (highly human-like condition) and a mechanized robot with no humanoid characteristics (machine-like condition; see Fig. 1). The scenario and variables measured are the same as in Study 4 (intention to acquire the medicine: Cronbach’s α = 0.80, social judgment: Cronbach’s α = 0.94, embarrassment manipulation check: Cronbach’s α = 0.97). The highly embarrassing product is again an STD medication and the less embarrassing product is an ear infection medication. We assessed the extent to which the service provider seemed robotic on a five-item, 7-point semantic differential scale (e.g., “artificial–lifelike”; Cronbach’s α = 0.88; Bartneck et al., 2009) and automated social presence with a four-item scale (e.g., “I can imagine the service provider to be a living creature”; Cronbach’s α = 0.92; 1 = “strongly disagree” and 7 = “strongly agree”; Čaić et al., 2019).

### Results

#### Manipulation checks

An ANOVA revealed that participants perceived the highly human-like service robot as more human-like than the machine-like robot (M_highlyhuman-like_ = 2.52, M_machine-like_ = 2.34; *F*(1,701) = 4.07, *p* = 0.044). In addition, participants felt significantly more embarrassed when acquiring STD antibiotics than when acquiring ear infection antibiotics (M_high_ = 4.81, M_low_ = 1.86; *F*(1,701) = 855.71, *p* < 0.001).

#### Automated social presence

An ANOVA revealed a main effect of type of service robot on automated social presence such that participants perceived significantly higher automated social presence for the anthropomorphic robot than for the machine-like robot (M_highlyhuman-like_ = 2.94, M_machine-like_ = 2.35; *F*(1,699) = 36.19, *p* < 0.001). The main effect of product type is not significant (*p* = 0.559). Interestingly, our analyses reveal a significant two-way interaction between service provider and product type (*F*(1,702) = 4.34, *p* = 0.038, η_p_^2^ = 0.01). Contrasts show that respondents encountering a highly human-like robot perceived marginally greater automated social presence when acquiring the embarrassing product than when acquiring the less embarrassing product (M_high_ = 3.07, M_low_ = 2.81; *F*(1,699) = 3.57, *p* = 0.059). However, perceived automated social presence was not significantly different when participants encountered the machine-like robot (M_high_ = 2.27, M_low_ = 2.42; *p* = 0.290).

#### Social judgment

An ANOVA revealed that respondents felt more socially judged by the anthropomorphic service than by the machine-like service robot (M_highlyhuman-like_ = 2.54, M_machine-like_ = 2.23; *F*(1,699) = 10.37, *p* = 0.001) and also when having to acquire the more embarrassing product (M_high_ = 2.61, M_low_ = 2.17; *F*(1,699) = 20.06, *p* < 0.001). The interaction between service provider and product type was significant (*F*(1,702) = 4.98, *p* = 0.026, η_p_^2^ = 0.01). Contrasts show that participants felt more socially judged when acquiring the more embarrassing product from a highly human-like service robot than from a machine-like service robot (M_highlyhuman-like_ = 2.87, M_machine-like_ = 2.34; *F*(1,699) = 14.75, *p* < 0.001, η_p_^2^ = 0.02). This was not the case when acquiring the less embarrassing product (M_highlyhuman-like_ = 2.22, M_machine-like_ = 2.12; *p* = 0.483).

#### Intention to acquire the medicine

An ANOVA revealed that respondents had stronger intentions to acquire the product from a machine-like service robot than from an anthropomorphic robot (M_highlyhuman-like_ = 4.92, M_machine-like_ = 5.19; *F*(1,699) = 9.34, *p* = 0.002), and stronger intentions to acquire the embarrassing product in general (M_high_ = 5.36, M_low_ = 4.75; *F*(1,699) = 48.08, *p* < 0.001). The service provider–product type interaction was marginally significant (*F*(1,702) = 2.74, *p* = 0.098). Contrasts show that intentions to acquire the embarrassing product were greater with a machine-like service robot than with a highly human-like service robot (M_highlyhuman-like_ = 5.15, M_machine-like_ = 5.56; *F*(1,699) = 11.03, *p* = 0.001, η_p_^2^ = 0.02). This was not the case when acquiring the less embarrassing product (M_highlyhuman-like_ = 4.69, M_machine-like_ = 4.82; *p* = 0.321).

#### Moderated serial mediation

To further test whether robot anthropomorphism and greater automated social presence may backfire when consumers fear social judgment, we conducted a moderated serial mediation analysis, in which the service robot served as the independent variable (machine-like robot = 0, highly human-like robot = 1), intention to acquire the medicine as the dependent variable, automated social presence as the stage-one mediator, social judgment as the stage-two mediator, and product embarrassment (low = 0, high = 1) as the moderator (Model 85; Hayes, 2018).

The results show that consumers are more likely to acquire an embarrassing product from a machine-like service robot than from a highly human-like service robot owing to perceived lower automated social presence and less social judgment by the robot. The analysis reveals a significant indirect effect through automated social presence and social judgment as serial mediators for both the embarrassing product (indirect effect = −0.09, SE_*b*_ = 0.02, 95% CI [−0.14, −0.05]) and the less embarrassing product (indirect effect = −0.04, SE_*b*_ = 0.02, 95% CI [−0.08, −0.01]). Importantly, the difference between the conditional indirect effects is significant (index of moderated mediation = −0.05, SE_*b*_ = 0.02, 95% CI [−0.10, −0.001]), showing that differences in robot appearance have stronger effects on automated social presence and social judgment when the product is embarrassing. In particular, we find that a highly human-like robot is perceived as having a higher level of automated social presence, which is strengthened when the consumer is acquiring an embarrassing product (b = 0.80, *p* < 0.001) compared to a less embarrassing product (b = 0.39, *p* = 0.006). This perceived automated social presence led to stronger feelings of being socially judged (b = 0.53, *p* < 0.001), which in turn negatively influenced the intention to acquire the embarrassing product from an anthropomorphic service robot instead of a machine-like service robot (b = −0.29, *p* < 0.001).

### Discussion

Study 5 extended the previous studies in two ways. First, robot anthropomorphism is associated with higher automated social presence, which makes people feel more judged. A less anthropomorphic robot therefore facilitates consumers’ acquisition of an embarrassing product. Second, results provided evidence that automated social presence plays a mediating role in this relationship and predicts feelings of social judgment, which in turn decreased the intention to acquire the medicine. Study 5 finds support for H3 and additionally reveals that the greater perceptions of automated social presence triggered by a highly anthropomorphic robot are strengthened if an embarrassing product is being acquired. Interestingly, we find a main effect of product type—intentions were higher to acquire the highly embarrassing (vs. less embarrassing) product, possibly owing to the potentially higher importance of buying STD antibiotics, whereas an ear infection may be treated with a home remedy.

## General discussion

In recent years, the use of service robots in organizational frontlines has accelerated (International Federation for Robotics, 2019; Wirtz et al., 2018), and robot receptionists, robot delivery, and robot assistants are expected to continue to replace human frontline services. Given that robotization is in full swing globally, we deliver insights on what is needed to help consumers accept being served by a robot.

Prior work predominantly shows that consumers prefer human service providers over service robots because robots can trigger negative feelings such as eeriness and being threatened (Čaić et al., 2019; Mende et al., 2019). We provide an important boundary condition to these findings and show that consumers react more positively to robotic service providers if human presence is the source of potential negative feelings. We focus on service encounters where human social presence causes perception of social judgment, such as when consumers need to acquire embarrassing products, are faced with criticism, or are confronted with their own mistakes (Grace, 2009; Higuchi & Fukada, 2002; Miller, 1996; Miller & Leary, 1992).

Building on social identity theory (Edelmann, 1987; Miller & Leary, 1992), we expected that in such embarrassing situations, human social presence would make people concerned for how they are appraised by others and fear social judgment by others, leading to less favorable attitudes toward the service provider and avoidance reactions such as not acquiring the embarrassing product or not accepting an alternative offer from the same service provider. In five studies, including a lab study with actual human–robot interactions and a field study in a real advertising context, we show that when social presence is automated by a service robot, anxiety about social judgment decreases, helping consumers to overcome reluctance to engage in embarrassing situations. Our findings are in line with a recent study showing that robots reduce feelings of embarrassment (Pitardi et al., 2021), yet importantly extends that study’s results to an array of managerially relevant outcomes, such as the propensity to acquire an embarrassing product or accept an alternative offering from the same service provider. While embarrassing products may be purchased online or via self-checkouts (Krishna et al., 2019; Jackson et al., 2014), embarrassing social service interactions as featured in our studies can arise unexpectedly, such as when the service provider confronts a customer with her own mistakes (Grace, 2009).

Given that literature on service robots emphasizes the crucial role of anthropomorphism in how people respond to robots (Mende et al., 2019), we also explore whether differences in robot anthropomorphism affect the extent to which consumers experience a robot as a social entity able to judge them. Although an anthropomorphic robot is perceived as more enjoyable to interact with and more trustworthy than a purely mechanized robot (Broadbent et al., 2013; van Pinxteren et al., 2019), consumers can respond more favorably to a service robot that is less human-like (e.g., Mende et al., 2019). In an embarrassing situation, encountering a highly human-like (vs. a machine-like) robot results in greater consumer awareness of the social presence of another entity, which increases the perception of social judgment—making consumers as reluctant to acquire the product as with a human service provider.

In sum, although customers’ acceptance of robots depends on social–emotional elements such as perceived social presence (Heerink et al., 2008; van Doorn et al., 2017; Wirtz et al., 2018), our results indicate that service robots can be perceived as more judgmental when their anthropomorphism and therewith their automated social presence increase. Importantly, our findings are in line with and extend work from the medical domain that a human-like robot evoked stronger feelings of embarrassment than a technical box during a medical examination (Bartneck et al., 2010) and that patients share more potentially embarrassing information with a machine-like robot (Kiesler et al., 2008; Złotowski et al., 2015).

### Theoretical contribution

Although a large body of research has examined the design and functionality of robots, studies of how users perceive frontline service robots in real service environments are limited and further work is much needed (Jörling et al., 2019). Importantly, we show a key boundary condition to earlier work, which cautions that consumers are reluctant to engage with service robots (Čaić et al., 2019; Mende et al., 2019). Specifically, we find that consumers are more accepting of service robots in situations where human presence is the source of negative emotions and triggers embarrassment. Embarrassment is a powerful emotion that can influence consumers in various meaningful ways (Krishna et al., 2015). Since consumers are inclined to avoid awkward service experiences or embarrassing products (Blair & Roese, 2013; Grace, 2007), service robots are a viable alternative that can reduce feelings of social judgment. We bring together two heretofore distinct literature streams—that on embarrassment and social judgment (Dahl et al., 2001; Grace, 2009) and that on service robots and technology (Huang & Rust, 2017, 2018; van Doorn et al., 2017)—and show how social identity theory affects people’s reactions to robots.

Extending prior work on robot anthropomorphism (Bartneck et al., 2010; Kim et al., 2019; Mende et al., 2019; van Pinxteren et al., 2019), we demonstrate the important role of a service robot’s anthropomorphic appearance in shaping perceptions of automated social presence (van Doorn et al., 2017). In addition, we add to the scarce knowledge on connecting the construct of automated social presence to downstream service outcomes (Čaić et al., 2019). While automated social presence of the robot may be beneficial in many situations, such as when developing trust (Wirtz et al., 2018), we caution that it backfires when consumers fear social judgment (Dahl et al., 2001; Miller & Leary, 1992).

### Managerial and practical implications

As the prevalence of robots in service settings increases, organizations that want to employ service robots need to understand consumers’ attitudes and behaviors toward them. Successful incorporation of robots into customer service is a significant challenge for most organizations. Firms need actionable guidance on how, when, and to what extent service robots should be adopted and how to use AI to engage customers in a more systematic and strategic way (Huang & Rust, 2020). Our findings have several practical managerial implications.

First, although studies have shown that disclosing AI before the machine–customer interaction significantly reduces the likelihood of purchase (Luo et al., 2019), managers worried about potential negative effects of service robots should be aware that in embarrassing situations, consumers can prefer robots to human employees. Accordingly, firms should not hesitate to use robots in service settings that consumers may experience as awkward and uncomfortable, thereby exploiting the advantages service robots may offer in customer interactions (Davenport et al., 2020).

Second, organizations need to pay attention to the anthropomorphic design of the robot, which may magnify feelings of negative social judgment. On the basis of our findings, we advise managers to make sure that service robots do not have an overly human-like resemblance. Very lifelike robots typically evoke eeriness and negative feelings (Mori et al., 2012) and are experienced as more judgmental because consumers perceive a greater automated social presence. Accordingly, a more technical-looking robot or a robot with only a few human characteristics, such as the robot “Pepper,” will be a more prudent option because the overall service experience will be more positive and will likely increase customer satisfaction.

Third, the implications of our work are broad and can extend to other situations where people may fear human social judgment. For example, consumers who seek healthcare may avoid discussing potentially embarrassing issues with their service providers, a behavior that has important economic, social, and health consequences at both the individual and the societal level (McCambridge & Consedine, 2014). Employing robots in elder care can mitigate feelings of shame and dependency, such as feeling burdensome to others (Grootegoed et al., 2013). In addition, service robots may be effective in financial services when dealing with sensitive personal information (Miao et al., 2021).

### Limitations and future research

This study has some limitations that offer opportunities for future research. First, in our Facebook advertising study we considered only click-through rates. It would be interesting to look at actual purchasing behavior and thus a more outcome-driven marketing metric such as cost per acquisition (CPA), which indicates the costs to get a customer to complete a specific action (e.g., a sale). Second, the generalizability of our results is limited to service encounters with no social entities present other than the service provider. Although as a means of behavioral coping with public embarrassment customers try to be alone or around others who would ignore them (Dahl et al., 2001; Lau-Gesk & Drolet, 2008), future work could address the effect of the physical environment in which a social entity is present but not involved with the customer in any way, especially as previous literature has indicated that noninteractive social situations can affect the service experience and service satisfaction (Argo et al., 2005; He et al., 2012).

A potentially interesting future research direction would be to examine whether our effects persist over multiple service encounters with a frontline service robot, given that we study first-time customer–robot service encounters. As embarrassment makes people feel uncomfortable, nervous, and flustered (Miller & Leary, 1992), the thought of the imminent embarrassing service encounter may already induce stress and evoke negative feelings. If consumers know in advance that they will encounter a service robot in a store, they are free from anticipating or experiencing embarrassment.

## Supplementary information

ESM 1 (DOCX 3.85 MB)

## Footnotes

1 Given limited access to the participant pool, we collected responses for Study 1 from all students who signed up to complete this lab session (rather than determining an a priori sample size; Kupor & Laurin, 2020). A sensitivity power analysis in G*Power (v3.1; Faul et al., 2009) indicated that this sample size provided 80% power to detect a medium-sized effect (w = 0.28) for the difference between conditions.

2 We determined the number of Facebook users on the basis of an estimated test power of 80% (Mookerjee et al., 2021).

3 A priori power analysis using G*Power (power of 0.80 and an alpha error probability of 0.05) yielded a minimum sample of 351 to detect a small to medium-sized two-way interaction effect (f = 0.15) between service provider and embarrassment. Given that we excluded participants who failed our attention checks (regarding human vs. robot service provider and the situation/product that was described), we overrecruited by about 15% and requested samples of 405 respondents for Studies 3 and 4, but sometimes received more/fewer responses because participants started the study simultaneously. Given that the differences between robots with varying levels of anthropomorphism are expected to be smaller than differences between a robot and a human, we seek adequate power to detect a small interaction effect (f = 0.10) in Study 5. While the required sample size was 787, financial constraints allowed us to collect only 709 responses.

4 A binary logistic regression with high (vs. low) embarrassment situation as independent variable on the choice between a robot vs. human service provider revealed that participants are more likely to click on the website with a service robot when the service is considered to be highly embarrassing (Choice_robot_ = 64.7%, Choice_human_ = 35.3%), whereas the opposite is true when the service is perceived to be less embarrassing (Choice_robot_ = 44%, Choice_human_ = 56%; Wald χ^2^ = 4.30, *p* = 0.038, η^2^ = 0.21).
