# Supplementary material for: Robots do not judge: service robots can alleviate embarrassment in service encounters
Source: J Acad Mark Sci. 2022 Apr 20:1–18. Online ahead of print. doi: 10.1007/s11747-022-00862-x (PMC9019535; doi:10.1007/s11747-022-00862-x)
Supplement: Supplementary file 1 — (DOCX 3.85 MB) [file 11747_2022_862_MOESM1_ESM.docx]

**Web Appendix**

**“Robots do not judge: Service robots can alleviate embarrassment in service encounters”**

This document has three main sections. In Section 1, we have disclosed all materials and measures used in our experiments. Information is presented according to the order of mention in the article. In Section 2, we have provided further information regarding the Facebook field study. In Section 3, we have provided additional data analyses, including the results of our robustness tests.

**SECTION 1: EXPERIMENTAL PROCEDURES AND MATERIALS**

**Study 1**

1. **Embarrassment manipulation**

Participants were randomly assigned to one of the two embarrassment conditions: *high* or *low* embarrassment. They were first asked to rate the product embarrassment of three products; specifically, participants in the *high embarrassment* condition rated a sex toy, a chlamydia self-test, and hemorrhoid cream, whereas those in the *low embarrassment* condition rated a fitness massage ball, a COVID-19 self-test, and hand cream. To measure product embarrassment as a manipulation check, we adapted a 7-point Likert scale from previous research (Dahl et al., 2001), consisting of four statements (1 = strongly disagree, 7 = strongly agree):

| - - - 1. Buying the [insert product] makes me feel embarrassed. |
| --- |
| - - - 1. Buying the [insert product] product makes me feel ashamed. |
| - - - 1. Buying the [insert product] product makes me feel uncomfortable. |
| - - - 1. Buying the [insert product] product makes me feel awkward. |

On the next page, participants in the *high embarrassment* condition read the following text:

In the following, please rank all three products on a scale according to how embarrassing it would be for you to buy them. The product you are most embarrassed to buy should be ranked as 1. The product least embarrassing to you should be ranked as 3. Please write the number in the box left to the text. You can assign every value only once.
The products are:


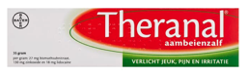
Sex toy             Chlamydia test              Hemorrhoid cream   

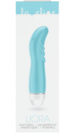
            
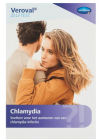


Participants in the *low embarrassment* condition read the following text:

In the following, please rank all three products on a scale according to how embarrassing it would be for you to buy them. The product you are least embarrassed to buy should be ranked as 1. The product most embarrassing to you should be ranked as 3. Please write the number in the box left to the text. You can assign every value only once.

The products are:

Fitness massage ball              Corona self-test                   Hand cream  

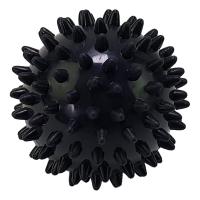
            
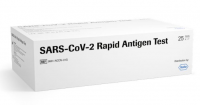
           
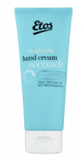


1. **Choice of service provider**

After participants have ranked the product, they read the following text:

You chose the [insert product].

In the next step, you have to acquire the [insert product]. Please read the following instructions carefully:

1. There are two locations where you could get a free sample of a [insert product], which are next to the entry of the research lab. In one location, you will encounter a human service provider who will hand you the product. In the other location, there is a service robot present who would give you the product. Both service providers do not know which product you chose and therefore you have to tell them. No one else except the service provider is in each room.
             
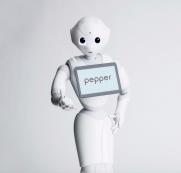
                 
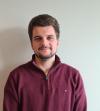


2. The service provider will hand you the product that you have to take back to your cubicle to proceed with the survey. To ensure that no one else knows which product you chose, you can put your product into a paper bag to carry it to your cubicle. You will find paper bags in both locations.

3. Both locations have a big sign on the door such that you know where to go. The room in which the human service employee is present is room A. The room in which the service robot is present is room B. 
At which location would you prefer to acquire the [insert product]?

| O Room A, from the human service employee |
| --- |
| O Room B, from the service robot |

On the next page, participants were instructed to now acquire the product that they have previously chosen to be most embarrassing (*high embarrassment* condition) or least embarrassing (*low embarrassment* condition):

Now please leave your cubicle to acquire the [insert product] that you have previously chosen.

Go to the entry hall of the research lab. There you will see the two locations, the signs on the doors clearly indicate which one is room A and room B.
       
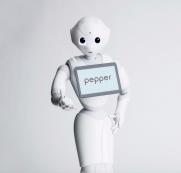

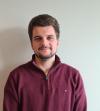


Remember, the human employee is in room A and the service robot is in room B. No one else will be present in either location and only you know which product you have to get.


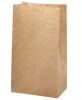
 After you got the [insert product], please use one of the paper bags to ensure that no one sees the product. Then, return with the product in the bag to your cubicle to continue with the study.

Photos of the locations in which the service provider was present can be found below:


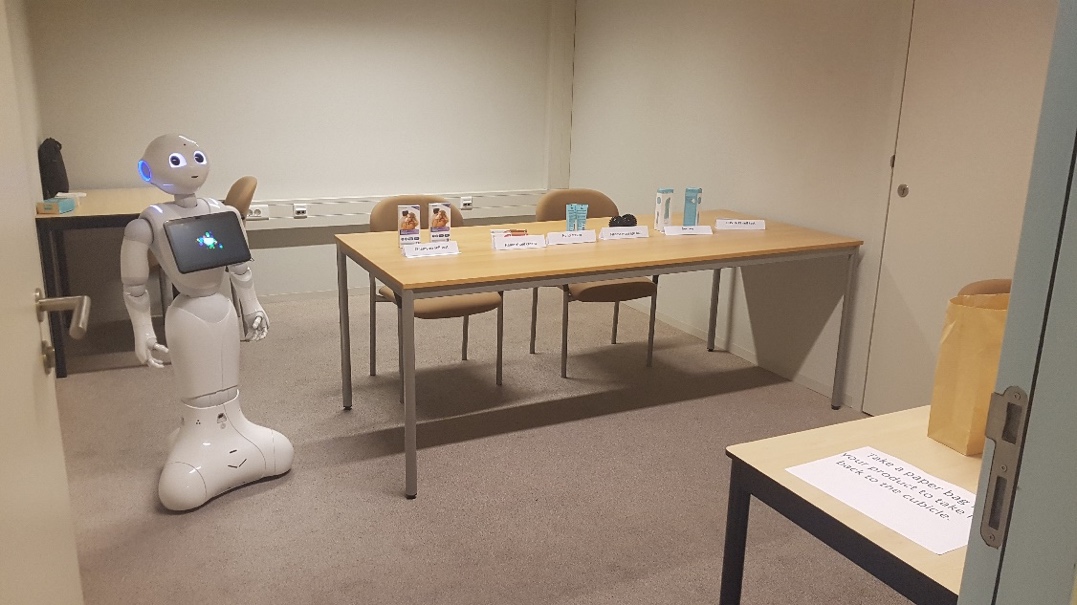


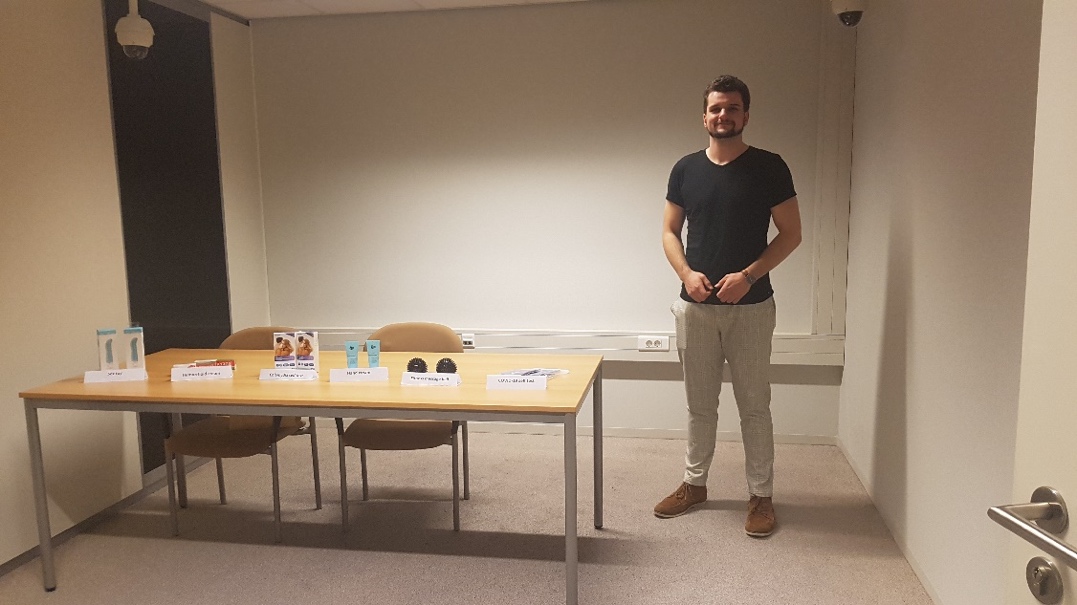


1. **Intentions to choose the robot vs. human service provider**

Before the participants were acquiring the chosen product from either of the service providers, we measured their intentions of choosing the robot vs. human. We used a four-item, 7-point semantic differential scale:

| 1. How likely are you to get the [insert product] from the human service provider or the service robot? |
| --- |
| 1. I intend to gather the [insert product] from the … service provider. |
| 1. If I had to do it over again, I would make the same choice and buy the [insert product] from the … service provider. |
| 1. The … service provider is a good option to get the [insert product]. |

1. **Covid-19 concerns**

Given the setup of our lab study, we controlled for COVID-19 concerns. Participants were asked about COVID-19 concerns at the end of the survey. As measurement, we used a 3-item scale, which participants responded to on 5-point scales (1 = none at all, 5 = a great deal):

| - - - 1. How much did the coronavirus (COVID-19) influence your choice of service provider to get the product? |
| --- |
| - - - 1. How much did the coronavirus (COVID-19) influence your responses to this study? |
| - - - 1. How much were you worried about the coronavirus (COVID-19) when completing this study? |

**Study 2**

1. **Embarrassment manipulation**

The ads were displayed on Facebook as sponsored posts.

In the *high embarrassment condition*, the ad showed the following headline:

Check your fat percentage! Consult our robot (human) advisor to prevent overweight and obesity.

Have a chat online about your health and body composition.

In the *low embarrassment condition*, the ad showed the following headline:

Check your fat percentage! Consult our robot (human) advisor to assess your fitness and nutritional diet.

Have a chat online about your health and body composition.

1. **Service provider manipulation**

Participants were randomly exposed to an ad showing either the *human* or *robot* service provider.

In the *robot* condition, this image appeared:


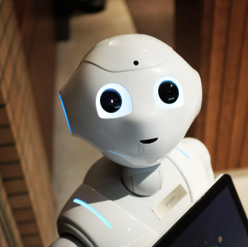


In the *human* condition, this image appeared:


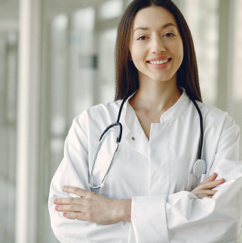


Once respondents clicked on one of the ads, they were redirected to a website on which we debriefed them (see Section 2 for the URLs).

**Study 3**

1. **Embarrassment manipulation**

Participants were randomly assigned to either the *high* or *low embarrassment* condition and were asked to read a scenario relating to the condition.

Participants in the *high embarrassment* condition received the following description:

Imagine you want to go out for dinner. You head for a new restaurant. Upon arrival at the restaurant, you see a host welcoming you at the door. The host manages the public part, or the "front of the house," of the restaurant and acts as a doorman.
The host tells you that unfortunately, you are not allowed to enter the restaurant without a reservation.

[page break]

On the next page, you will be shown a picture of the restaurant where you would like to go for dinner. There the host is present, who tells you that you are not allowed to enter because you have no reservation.

Participants in the *low embarrassment* condition received the following description:

Imagine you want to go out for dinner. You head for a new restaurant. Upon arrival at the restaurant, you see a host welcoming you at the door. The host manages the public part, or the "front of the house,” of the restaurant and acts as a doorman.
The host tells you that unfortunately, you cannot enter because the restaurant is full.

[page break]

On the next page, you will be shown a picture of the restaurant where you would like to go for dinner. There the host is present, who tells you that you cannot enter because no tables are available.

1. **Service provider manipulation**

Participants were randomly assigned to either the *human* or *robot* service provider condition. In both conditions, participants were shown the following sentence, which was accompanied by an image:

Please take a moment to closely look at the picture below.

In the *robot* condition, this image appeared:


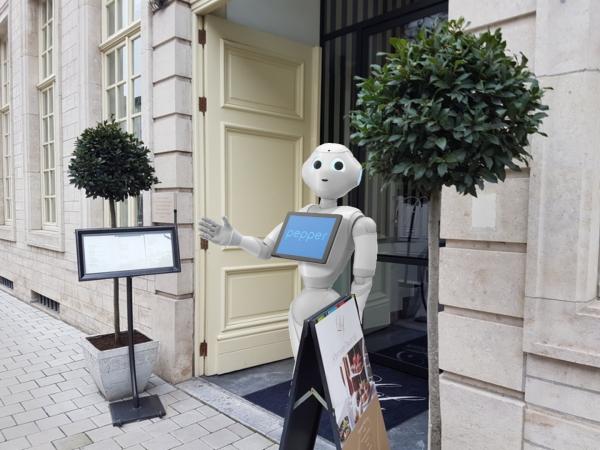


In the *human* condition, this image appeared:


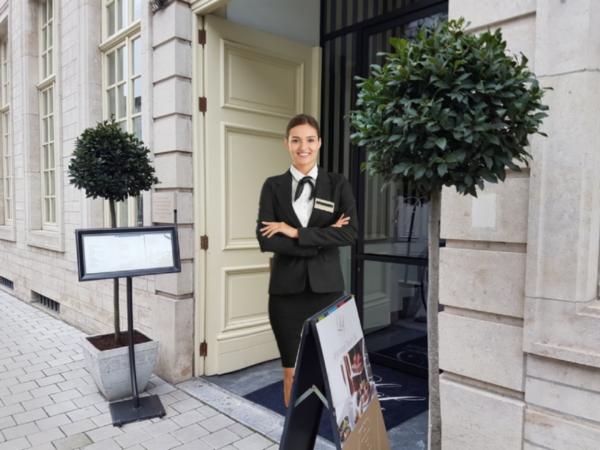


1. **Likelihood to accept alternative offering**

Participants were shown the following text before the dependent variable was measured:

Imagine the host mentions that the restaurant on the other side of the street belongs to the same owners. The food is just as delicious, yet no reservation is required to enter [high embarrassment condition] / and there are enough tables available [low embarrassment condition].

To measure our dependent variable, we used a scale consisting of four statements, which participants responded to on 7-point scales (1 = strongly disagree, 7 = strongly agree):

| - - - 1. Eating at the other restaurant seems to be a good alternative. |
| --- |
| - - - 1. I will go to the other owner’s restaurant instead because there are tables available / no reservation required. |
| - - - 1. I intend to go to a completely different restaurant. (R) |
| - - - 1. Owing to the encounter with the host, I would prefer to go somewhere else and not to the other recommended restaurant. (R) |

**Study 4**

1. **Embarrassment manipulation**

Participants were randomly assigned to either the *high embarrassment* or *low embarrassment* condition and were asked to read the following text.

Participants in the *high embarrassment* condition saw the following:

Please imagine that you have been to the doctor and you got a prescription for antibiotics for your sexually transmitted disease (STD). Now you go to the pharmacy to collect the antibiotics.

[page break]

On the next screen you will be shown a picture of the pharmacy where you can collect the STD antibiotics. There is a service provider present in the pharmacy that gives the antibiotic to you.

Participants in the *low embarrassment* condition saw the following:

Please imagine that you have been to the doctor and you got a prescription for antibiotics for your ear infection. Now you go to the pharmacy to collect the antibiotics.

[page break]

On the next screen you will be shown a picture of the pharmacy where you can collect the ear infection antibiotics. There is a service provider present in the pharmacy who gives the antibiotic to you.

1. **Service provider manipulation**

Participants were randomly assigned to either the *robot* or *human* service provider condition. In both conditions, participants were shown the following sentence:

Please take a moment to closely look at the picture below. Imagine you are standing in front of the pharmacy.

On the same page, participants saw an image of the pharmacy.

In the *robot* condition, these images were displayed:


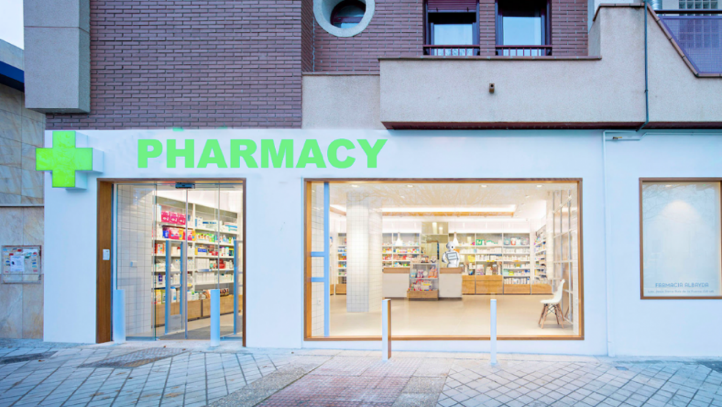

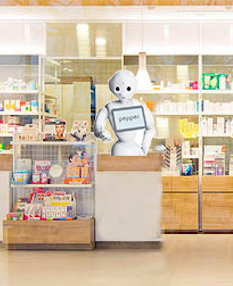


In the *human* condition, these images were displayed:


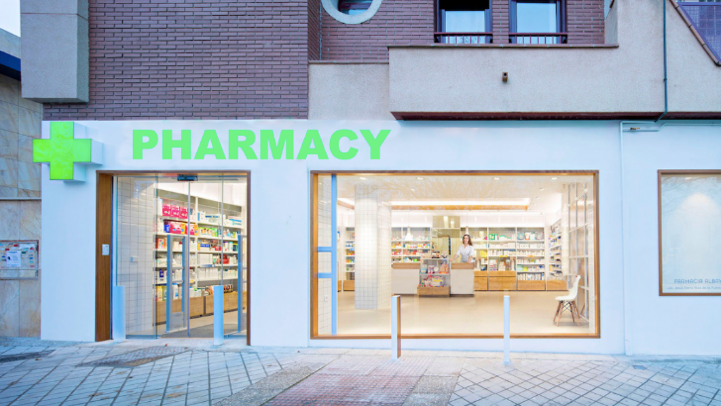

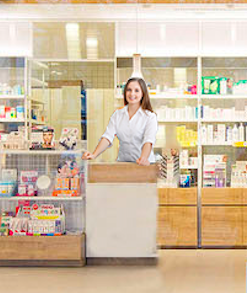


1. **Measuring social judgment**

To measure social judgment, we used the social evaluation scale from previous research (Manstead & Semin, 1981; Miller, 1996; Semin & Manstead, 1981). This scale consists of six items, which participants rated using 7-point scales (1= strongly disagree, 7= strongly agree):

| 1. I was concerned that I was being evaluated in an undesirable way by the service provider. |
| --- |
| 1. I was worried about how the service provider would evaluate me. |
| 1. I was anxious that I would fail to create the desired impression on the service provider. |
| 1. I was anxious that the service provider might have a bad impression of me. |
| 1. I was afraid that the service provider around me might think ill of me. |
| 1. I was concerned that the service provider would think badly of me. |

1. **Intention to acquire the medicine**

We used a four-item, 7-point scale to measure our DV, which was the intention to enter the pharmacy and acquire the medicine (1= strongly disagree, 7= strongly agree):

| 1. How likely are you to get your antibiotics at this pharmacy instead of going to another pharmacy? |
| --- |
| 1. I intend to gather the antibiotics at this pharmacy from this service provider. |
| 1. If I had to do it over again, I would make the same choice. |
| 1. This pharmacy is a good option to get the antibiotics. |

**Study 5**

1. **Embarrassment manipulation**

This manipulation is the same as in Study 4.

1. **Service provider manipulation**

Participants were randomly assigned to one of the two service provider conditions—*highly human-like* *robot* or *machine-like robot*—and were shown the following sentence:

Please take a moment to closely look at the picture below. Imagine you are standing in front of the pharmacy.

On the same page, participants saw an image.

In the *highly human-like robot* condition, these images were displayed:

**
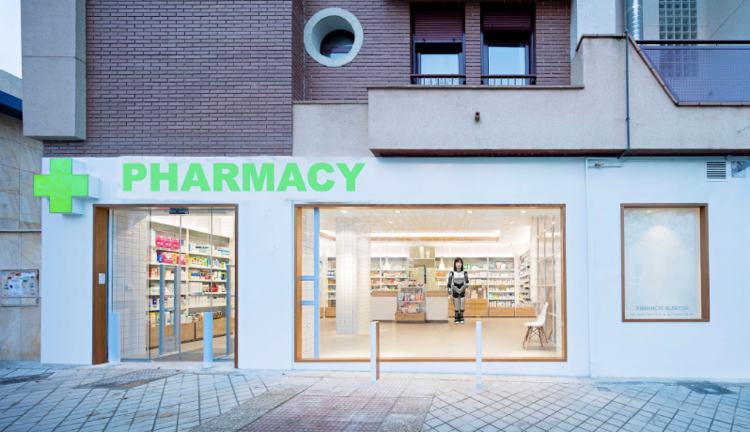
**
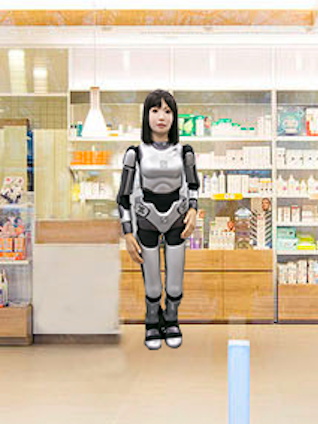


In the *machine-like robot* condition, these images were displayed:

**
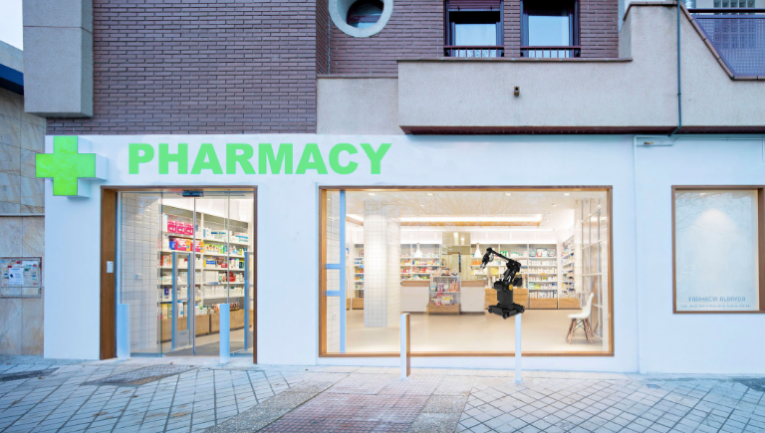
**
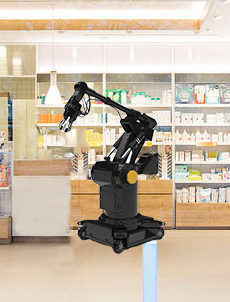


1. **Measuring automated social presence**

We assessed automated social presence with a scale from Čaić et al. (2019). This scale consists of four statements, which participants rated using 7-point scales (1= strongly disagree, 7= strongly agree):

| 1. I can imagine the service provider to be a living creature. |
| --- |
| 1. When interacting with the service provider I felt like I was talking with a real person. |
| 1. The service provider seems to have real feelings. |
| 1. I felt like the service provider was actually looking at me throughout the conversation. |

1. **Measuring social judgment**

We used the same scale as in Study 4.

1. **Intention to acquire the medicine**

We used the same scale as in Study 4.

1. **Robot anthropomorphism**

We assessed the extent to which the service provider seemed robotic on a 5-item, 7-point semantic differential scale from Bartneck et al. (2009):

| 1. Fake vs. Natural |
| --- |
| 1. Machine-like vs. Human-like |
| 1. Unconscious vs. Conscious |
| 1. Artificial vs. Lifelike |
| 1. Moving rigidly vs. Moving elegantly |

**SECTION 2: FURTHER INFORMATION REGARDING THE FACEBOOK ADS**

This section provides information about the descriptive statistics (Table 3) and ad specifications (Table 4) regarding Study 2.

**Table 3.** Descriptive statistics

| Condition | Reach | Clicks | CTR |
| --- | --- | --- | --- |
| Human, low embarrassment | 3,034 | 22 | 0.73 |
| Human, high embarrassment | 3,239 | 24 | 0.74 |
| Robot, low embarrassment | 3,102 | 29 | 0.93 |
| Robot, high embarrassment | 3,243 | 41 | 1.26 |
| Total | 11,815 | 116 |  |

CTR = click-through rate (clicks / reach x 100)

**Table 4.** Facebook Ad specifications

| **Special ad categories** | No categories declared |
| --- | --- |
| **Campaign Details** | *Buying Type:* Auction  *Objective*: Traffic |
| **Create A/B Test** | Disabled |
| **Campaign Budget Optimization** | Off |
| **Traffic** | Website |
| **Dynamic Creative** | Off |
| **Budget & Schedule** | €50.00 (€22.54 due to account spend limit) per day  Start date: Jan 23, 2022, 11:14 AM (Amsterdam Time) |
| **Audience Details** | *Location*: United Kingdom  *Age*: 18-65+  *Gender*: All genders  *Detail targeting*: All demographics, interests and behaviors  *Detailed targeting expansion*: Off  *Languages*: All languages  *Connections*: All people |
| **Placements** | Manual placements  *Devices*: All devices  *Platforms*: Facebook  *Placements*: Facebook News Feed |
| **Optimization & Delivery** | *Optimization for ad delivery*: Link Clicks  *Cost-Control*: None  *When you get charged*: Impression  *Delivery Type*: Standard |
| **Ad Setup** | Single image |
| **Ad Creative** | Media (Robot): Media (Human):  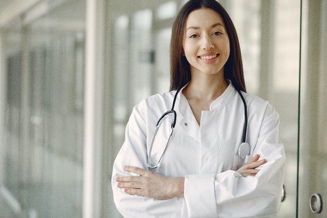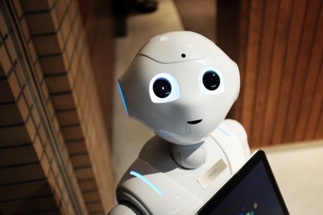  *Primary text (high embarrassment):* Check your fat percentage! Consult our robot advisor to prevent overweight and obesity. Have a chat online about your health and body composition.  *Primary text (low embarrassment):* Check your fat percentage! Consult our robot advisor to assess your fitness and nutritional diet. Have a chat online about your health and body composition.  *Headline*: Check your fat percentage!  *Description*: Have a chat online about your health and body composition.  *Optimize text per person*: Disabled  *Destination*: Website  *Website URL (robot):* https://sites.google.com/view/researchlabfebrobot  *Website URL (human):* https://sites.google.com/view/researchlabfebhuman  *Display link*: None/empty  *Class to action*: Contact us. |
| **Tracking** | None |

**SECTION 3: ROBUSTNESS CHECKS**

In our experiments, we found that embarrassment moderates the relationship between service provider type and the dependent variable (results differ according to the study). To assess the robustness of our results, we conducted several regressions in which we controlled for participants’ demographic characteristics (age and gender) and compared this model (Model 2) with our basic model (Model 1), which included only the main (manipulated) variables, service provider type, and embarrassment. The results are displayed in Tables 5–8 and show that the interaction between service provider and embarrassment remained significant even after controlling for demographic variables, confirming the robustness of our main findings.

**Table 5.** Choice of service provider serves as the dependent variable (Study 1)

|  | Model 1 | | |  | Model 2 | | |
| --- | --- | --- | --- | --- | --- | --- | --- |
| Variable | B | SE | Wald |  | B | SE | Wald |
| Intercept | 1.10 | 0.33 | 10.86** |  | -0.69 | 1.55 | 0.20 |
| Embarrassment | -1.85 | 0.45 | 16.90*** |  | -1.93 | 0.49 | 15.82*** |
|  |  |  |  |  |  |  |  |
| Age |  |  |  |  | 0.10 | 0.07 | 2.15 |
| Gender |  |  |  |  | -0.65 | 0.48 | 1.89 |
| COVID-19 concerns |  |  |  |  | -0.00 | 0.36 | 0.00 |

Regression coefficients are unstandardized. Service provider was coded 1 = robot, and 0 = human. Embarrassing product was coded 1 = yes, and 0 = no. Statistics are rounded to two digits after the decimal point.

† *p* < 0.10, ∗ *p* < 0.05, ∗∗ *p* < 0.01, ∗∗∗ *p* < 0.001*.*

**Table 6.** Likelihood to accept alternative offering serves as the dependent variable (Study 3)

|  | Model 1 | | |  | Model 2 | | |
| --- | --- | --- | --- | --- | --- | --- | --- |
| Variable | B | SE | t |  | B | SE | t |
| Intercept | 3.86 | 0.08 | 48.71*** |  | 3.69 | 0.13 | 27.91*** |
| Service provider | -0.13 | 0.11 | -1.15 |  | -0.15 | 0.11 | -1.32 |
| Embarrassment | -0.37 | 0.11 | -3.42*** |  | -0.37 | 0.11 | -3.42*** |
| Service provider × Embarrassment | 0.36 | 0.15 | 2.37* |  | 0.38 | 0.15 | 2.49* |
|  |  |  |  |  |  |  |  |
| Age |  |  |  |  | 0.00 | 0.00 | 0.55 |
| Gender |  |  |  |  | 0.12 | 0.07 | 1.56 |

Regression coefficients are unstandardized. Service provider was coded 1 = robot, and 0 = human. Embarrassing service situation was coded 1 = yes, and 0 = no. Statistics are rounded to two digits after the decimal point.

† *p* < 0.10, ∗ *p* < 0.05, ∗∗ *p* < 0.01, ∗∗∗ *p* < 0.001.

**Table 7.** Intention to acquire the medicine serves as the dependent variable (Study 4)

|  | Model 1 | | |  | Model 2 | | |
| --- | --- | --- | --- | --- | --- | --- | --- |
| Variable | B | SE | T |  | B | SE | t |
| Intercept | 3.89 | 0.08 | 47.73*** |  | 3.72 | 0.14 | 27.33*** |
| Service provider | -0.15 | 0.11 | -1.30 |  | -0.17 | 0.11 | -1.47 |
| Embarrassment | -0.39 | 0.11 | -3.48*** |  | -0.39 | 0.11 | -3.47*** |
| Service provider × Embarrassment | 0.42 | 0.16 | 2.67** |  | 0.44 | 0.16 | 2.78** |
|  |  |  |  |  |  |  |  |
| Age |  |  |  |  | 0.00 | 0.00 | 0.48 |
| Gender |  |  |  |  | 0.12 | 0.08 | 1.55 |

Regression coefficients are unstandardized. Service provider was coded 1 = robot, and 0 = human. Embarrassing product was coded 1 = yes, and 0 = no. Statistics are rounded to two digits after the decimal point.

† *p* < 0.10, ∗*p* < 0.05, ∗∗ *p* < 0.01, ∗∗∗ *p* < 0.001.

**Table 8.** Intention to acquire the medicine serves as the dependent variable (Study 5)

|  | Model 1 | | |  | Model 2 | | |
| --- | --- | --- | --- | --- | --- | --- | --- |
| Variable | B | SE | t |  | B | SE | t |
| Intercept | 4.85 | 0.09 | 55.69*** |  | 5.04 | 0.19 | 26.55*** |
| Service provider | -0.12 | 0.12 | -0.99 |  | -0.14 | 0.12 | -1.13 |
| Embarrassment | 0.75 | 0.12 | 6.07*** |  | 0.73 | 0.12 | 5.96*** |
| Service provider × Embarrassment | -0.29 | 0.17 | -1.66† |  | -0.25 | 0.17 | -1.47 |
|  |  |  |  |  |  |  |  |
| Age |  |  |  |  | 0.00 | 0.00 | 0.94 |
| Gender |  |  |  |  | -0.24 | 0.09 | -2.75** |

Regression coefficients are unstandardized. Service provider was coded 1 = highly human-like robot, and 0 = machine-like robot. Embarrassing product was coded 1 = yes, and 0 = no.

Statistics are rounded to two digits after the decimal point. † *p* < 0.10, ∗ *p* < 0.05, ∗∗ *p* < 0.01, ∗∗∗ *p* < 0.001.
